# Itch perception is reflected by neuronal ignition in the primary somatosensory cortex

**DOI:** 10.1093/nsr/nwab218

**Published:** 2021-12-03

**Authors:** Xiao-Jun Chen, Yan-He Liu, Ning-Long Xu, Yan-Gang Sun

**Affiliations:** Institute of Neuroscience, State Key Laboratory of Neuroscience, Center for Excellence in Brain Science and Intelligence Technology, Chinese Academy of Sciences, Shanghai 200031, China; University of Chinese Academy of Sciences, Beijing 100049, China; Institute of Neuroscience, State Key Laboratory of Neuroscience, Center for Excellence in Brain Science and Intelligence Technology, Chinese Academy of Sciences, Shanghai 200031, China; University of Chinese Academy of Sciences, Beijing 100049, China; Institute of Neuroscience, State Key Laboratory of Neuroscience, Center for Excellence in Brain Science and Intelligence Technology, Chinese Academy of Sciences, Shanghai 200031, China; University of Chinese Academy of Sciences, Beijing 100049, China; Shanghai Center for Brain Science and Brain-Inspired Intelligence Technology, Shanghai 201210, China; Institute of Neuroscience, State Key Laboratory of Neuroscience, Center for Excellence in Brain Science and Intelligence Technology, Chinese Academy of Sciences, Shanghai 200031, China; Shanghai Center for Brain Science and Brain-Inspired Intelligence Technology, Shanghai 201210, China

**Keywords:** itch, perception, primary somatosensory cortex, miniature two-photon microscopic imaging

## Abstract

Multiple cortical areas including the primary somatosensory cortex (S1) are activated during itch signal processing, yet cortical representation of itch perception remains unknown. Using novel miniature two-photon microscopic imaging in free-moving mice, we investigated the coding of itch perception in S1. We found that pharmacological inactivation of S1 abolished itch-induced scratching behavior, and the itch-induced scratching behavior could be well predicted by the activity of a fraction of layer 2/3 pyramidal neurons, suggesting that a subpopulation of S1 pyramidal neurons encoded itch perception, as indicated by immediate subsequent scratching behaviors. With a newly established optogenetics-based paradigm that allows precisely controlled pruritic stimulation, we found that a small fraction of S1 neurons exhibited an ignition-like pattern at the detection threshold of itch perception. Our study revealed the neural mechanism underlying itch perceptual coding in S1, thus paving the way for the study of cortical representation of itch perception at the single-neuron level in freely moving animals.

## Teaser

Miniature two-photon imaging of neuronal activity in free-moving mice reveals the perceptual coding of itch in the primary somatosensory cortex.

## INTRODUCTION

Itch represents a submodality of somatosensation, serving as an important protective mechanism. Although recent studies have started to reveal the mechanism of itch processing in the brain [1,2], the cerebral mechanism underlying the perception of itch, one of the most important and intriguing questions in the itch field, remains largely unknown. Early human studies using macroscopic brain imaging approaches, such as the positron emission tomography (PET) scan and functional MRI (fMRI), showed that many cortical regions, including the primary somatosensory cortex (S1), were activated by peripherally applied pruritic stimuli [3–6]. S1 has been proposed to encode the spatio-temporal and intensity aspects of itch sensation [7,8]. These functional imaging approaches have a relatively low temporal resolution, and do not reveal the neural dynamics underlying itch perception. Recently, Khasabov *et al*. examined the response of S1 neurons to pruritic stimuli with single-unit electrophysiological recording, and found that S1 neurons were indeed excited by pruritic stimuli [9]. However, this study was conducted in anesthetized animals that could not report the itch perception occurring periodically, which would have been reflected by the scratching behavior in awake animals. Similarly, most previous electrophysiological studies examining the coding mechanisms of itch in the brain or spinal cord were performed in anesthetized animals [10–13]. Thus, it remains to be determined whether and how S1 encodes information for itch perception, and this needs to be addressed in awake animals.

The transformation of sensory information into conscious perception has been an active research field in cognitive neuroscience [14,15]. Earlier studies trained animals to behaviorally report sensory perception, while using single-cell recording or population imaging to examine the potential roles of S1 or other cortical regions during the perceptual process. These studies have revealed diverse activity patterns of S1 in touch perception [16–18]. However, the animals in early studies all underwent varying degrees of training [19–21], which could in turn modify the response property of S1 neurons associated with the perceptual process [22,23], thus the intrinsic property of S1 neurons during sensory perception still remains unknown. It is critical to examine the perceptual coding capability of S1 using more natural sensory detection tasks, in which no learning is required for animals to report subjective perception.

In this study, by taking advantage of the natural behavior report of itch perception and a novel miniature two-photon microscope (mini-2P) [24], we investigated the coding of itch perception in S1 at single-cell resolution in free-moving mice responding to chemical itch. We also further deciphered the neural mechanism underlying itch perceptual detection in S1 by using an optogenetics-based approach, which allows for precise control of itch intensity.

## RESULTS

### *In vivo* two-photon calcium imaging in S1Tr of free-moving mice

**Figure 1.**
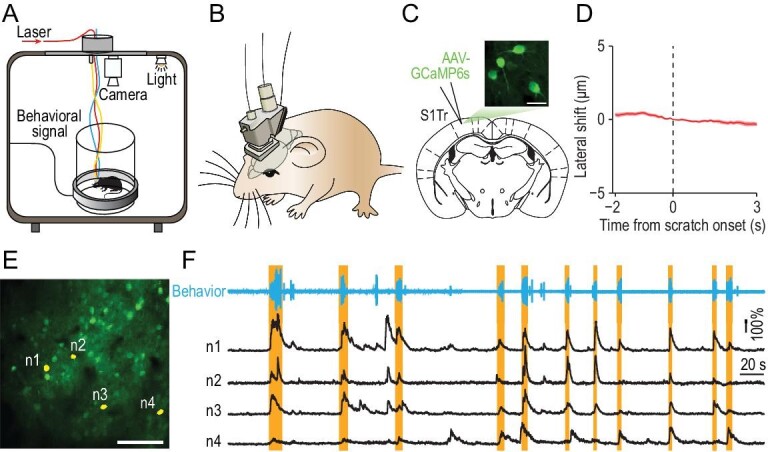
*In vivo* two-photon calcium imaging in S1Tr of free-moving mice. (A) Schematic of simultaneous calcium imaging and scratching behavior recording in free-moving mice. (B) Schematic of the mini-2P mounted on a mouse head. (C) Graph showing image of the layer 2/3 neurons labeled with GCaMP6s in S1Tr, acquired with the mini-2P from a free-moving mouse. Scale bar, 25 μm. (D) Lateral shift around the onset of scratching behavior in response to chloroquine during imaging. Shading represents SEM. (E) One example field of view (FOV) in an imaging session after intradermal injection of chloroquine. Scale bar, 100 μm. (F) Top: behavioral trace of one mouse during a 450-s period from an imaging session after intradermal injection of chloroquine. Bottom: calcium traces of four representative S1Tr pyramidal neurons in (E). Orange shading indicates periods of scratching.

To explore the representation of itch perception in mice, we measured the neuronal activity of the trunk region of S1 (S1Tr), which is required for processing pruritogen-evoked itch signals (Supplementary Fig. S1A–J), by recording calcium transients with the mini-2P (Fig. 1A and B). The advantage of the mini-2P is to allow animals to move freely, enabling mice to exhibit scratching behavior, as mice have difficulty scratching an itch under the head-fixed conditions required for traditional two-photon microscopes. We injected AAV-CaMKII-GCaMP6s [25] in S1Tr (Fig. 1C; Supplementary Fig. S1K) to express genetically encoded calcium indicator GCaMP6s, and implanted a chronic window above the injection site. Three weeks after the viral injection, we mounted the mini-2P on the heads of mice and recorded the calcium activity of pyramidal neurons in layer 2/3 of S1Tr (Fig. 1A and B; Supplementary Movie S1), with simultaneous recording of the scratching behavior using a magnetic induction method after intradermal injection of chloroquine [26]. For each imaging field, we recorded the calcium activity of hundreds of neurons with single-cell resolution (201 ± 59 neurons per field, five fields from five mice). The consistency of the fluorescence from identified cells showed a high stability (Supplementary Fig. S1L–O), with <5 μm lateral shift of the imaging field throughout the whole session, even during scratching induced by intradermal injection of chloroquine (Fig. 1D; Supplementary Fig. S1P). We found that S1Tr pyramidal neurons exhibited diverse activity patterns after pruritic stimulation, with some neurons displaying calcium transients that correlated with pruritogen-evoked scratching events (Fig. 1E and F). We analyzed the number and patterns of scratching events in animals with mini-2Ps as described earlier [27], and found that they were not significantly affected by the mini-2P mounted on the head (Supplementary Fig. S1Q–U).

### Representation of itch perception in S1

**Figure 2.**
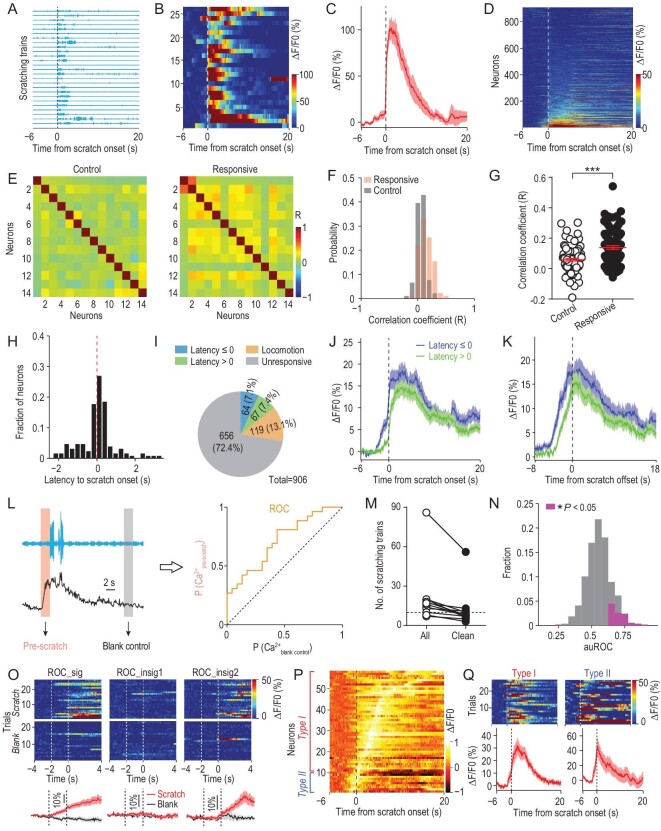
The primary somatosensory cortex encoded itch perception. (A) Scratching traces of an example mouse in response to intradermal injection of chloroquine aligned to the scratching onset of individual trains in an imaging session. Each row indicated one behavioral trial (train). (B) Heat map showing the calcium activity of one example neuron during the corresponding scratching trains in (A). (C) Averaged calcium trace of this neuron corresponding to all scratching trains. Shading represents SEM. (D) Heat map of all imaged neurons (*n* = 906) from five FOVs (5 mice) during chloroquine-induced scratching behavior. Each row indicated the averaged calcium activity of one neuron from all scratching trains in the chloroquine session. Neurons were rank-ordered by their response magnitude after the scratching onset. Vertical dashed line, scratching onset. (E) Pair-wise correlation matrix for randomly sampled control or responsive neurons from one FOV during the behaviorally quiet period without scratching after chloroquine injection. Neurons were rank-ordered by the correlation coefficient. (F) Distribution of pair-wise correlations from neurons in (E). (G) Quantification of correlations between the two groups of neurons from (F). Mann-Whitney test. ^***^P < 0.001. (H) Distribution of the response latency to the scratching onset of all responsive neurons (*n* = 131). (I) Composition of all imaged neurons in response to intradermal injection of chloroquine. (J) Averaged calcium traces of corresponding responsive neuron types. (K) Averaged calcium trace of different types of responsive neurons aligned to the scratching offset. (L) Schematic for the ROC analysis. The activity of the 2-s epoch before the scratching onset or during a behaviorally quiet period was used as the itch or blank control sample for ROC analysis. (M) The number of scratching trains in the chloroquine session before and after extracting the defined clean trains (*n* = 11 mice). Horizontal dashed line indicates 10 scratching trains. (N) Distribution of the area under the ROC curve (auROC) of 537 neurons from 4 mice. Neurons with significant discriminability after excluding locomotion-responsive neurons are shown in magenta. (O) Response patterns exemplified by three representative neurons in the ROC analysis. ROC_sig, neuron with high discriminability. ROC_insig, neurons not significantly discriminating two different trial types (scratch vs. blank control). (P) Normalized population activity of the neurons with high discriminability from ROC analysis in (N) (*n* = 58 neurons). Neurons are ordered by the peak time. Neurons showing sustained activity with peak occurring after the scratching onset are classified as type I, while those showing transient activity with peak occurring prior to the scratching onset are classified as type II. (Q) Left, one example type I neuron showing sustained activity before and after the scratching onset. Right, one example type II neuron showing transient activity before the scratching onset.

To exclude purely motor-related S1Tr neurons, we first aligned the activity of imaged neurons to the onset of locomotion events as indicated by non-itch hindlimb movements recorded by the magnetic induction method (Supplementary Fig. S2A), and found that ∼20.6% (187/906) of all imaged S1Tr neurons were activated near the locomotion onset (Supplementary Fig. S2B–D). We thus excluded these locomotion-activated neurons from further analysis. To examine the neural correlates of itch perception as indicated by scratching behavior, we aligned the activity of S1Tr pyramidal neurons to the onset of the scratching trains that had a minimal 10-s quiet period both before and after the scratching (Fig. 2A). These scratching trains were chosen to minimize the interference between different trains on the neuronal activity of S1, and were defined as ‘clean’ scratching trains (Methods). We found that 14.5% (131/906) of recorded neurons were reliably activated near the scratching onset, although the percentage of responsive neurons varied among the mice recorded (Fig. 2B–D; Supplementary Fig. S2E).

The responsive neurons exhibited high trial-by-trial reliability, and these neurons were widely distributed in the entire imaging field, exhibiting no spatial clustering (Supplementary Fig. S2F–H). However, the temporal correlation between responsive neurons was significantly higher than that between randomly sampled neurons during the behaviorally quiet period with no scratching detected (Fig. 2E–G; Supplementary Fig. S2I; Methods), suggesting that the S1Tr neurons responding to pruritic stimuli tend to exhibit synchronized activity.

The scratching behavior is likely to evoke other somatosensations such as touch or pain. However, neuronal activation associated with these sensations will only occur after the scratching onset. We thus analyzed the response latency of each neuron relative to the onset of scratching behavior, and found that a large fraction (64/131 or 48.9%) of responsive neurons were activated before the scratching onset (Fig. 2H–J; Supplementary Fig. S2J and K). Moreover, the responsive neurons decreased their activity immediately after the scratching offset (Fig. 2K; Supplementary Fig. S2L–P). These data indicate that the S1Tr neurons showing increased activity before the scratching onset are likely to represent itch signals in response to pruritic stimuli, while those activated after the scratching behavior onset presumably respond to scratching-induced touch/pain stimuli.

We next determined whether S1Tr neurons encoded the itch information that could predict the scratching behavior. We performed a receiver operating characteristic (ROC) analysis using the activity of recorded S1Tr neurons 2 s prior to the onset of scratching trains (Fig. 2L). In mice (*n* = 4) with a relatively large number (>10) of clean scratching trains (Fig. 2M), the pre-scratching activity of 10.8% (58/537) of all imaged S1Tr neurons could indeed discriminate scratching vs. non-scratching phases, indicating that these S1Tr neurons encoded sufficient information to predict the scratching behavior (Fig. 2N and O, left). We found that those S1 neurons failing to discriminate scratching vs. non-scratching phases displayed diverse response patterns near the onset of scratching, with 77.2% of them exhibiting no activation (Fig. 2O, middle), 11.8% of them exhibiting elevated activity before (Supplementary Fig. S2Q) and 11.1% of them exhibiting elevated activity after scratching onset (Fig. 2O, right). The neurons with high discriminability exhibited two response patterns based on their response peak time (Fig. 2P). Type I neurons (48/58 or 82.8%) showed a progressive increase in their activity that reached a peak after the scratching onset, suggesting that these putative itch-responsive neurons might also respond to other somatosensory inputs accompanying scratching (Fig. 2Q, left), consistent with the polymodal property of S1 neurons [9,28,29]. By contrast, type II neurons (10/58 or 17.2%) only exhibited transient activation that peaked before the scratching onset (Fig. 2Q, right), indicating that these neurons have a selective role encoding itch perception.

### Opto-itch evoked comparable responses in S1 as the chemical pruritogen

**Figure 3.**
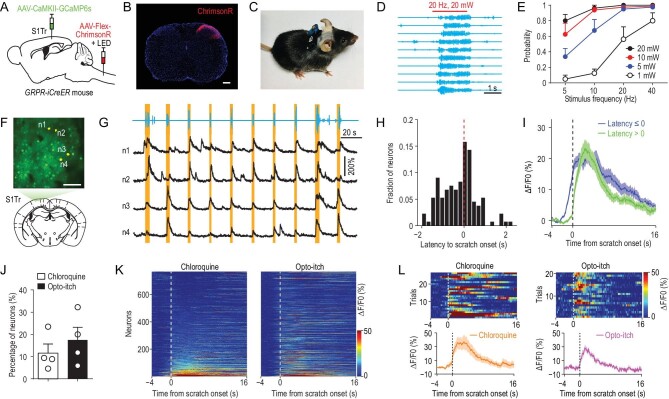
Opto-itch evoked comparable responses in the primary somatosensory cortex as the chemical pruritogen. (A) Schematic of the surgical preparation for simultaneous optogenetic stimulation of spinal GRPR^+^ neurons and calcium imaging in S1Tr. (B) Expression of ChrimsonR in GRPR^+^ neurons of the cervical dorsal spinal cord. Scale bar, 200 μm. (C) Picture of one mouse carrying a wireless receiver attached to the LED implanted above the spinal cord. (D) Example behavioral traces for scratching induced by optogenetic stimulation (630 nm, 20 Hz, 20 mW) of spinal GRPR^+^ neurons. Each row represents one trial. Red bar, opto-itch stimulation. (E) Quantification of the occurrence probability of scratching behavior in response to optogenetic stimulation of spinal GRPR^+^ neurons at different intensities (*n* = 8 mice). (F) An example FOV in the contralateral S1Tr following the surgery in (A) for simultaneous calcium imaging and opto-itch stimulation. Scale bar, 100 μm. (G) Top: behavioral trace of one mouse during a 210-s period of one imaging session with the opto-itch stimulation. Red bars, opto-itch stimulation (2 s, 5 Hz, 20 mW). Bottom: calcium traces of four representative S1Tr pyramidal neurons during this period. Orange shading indicates periods of scratching. (H) Distribution of the response latency to the scratching onset of all responsive neurons (*n* = 133 neurons) in the opto-itch session after excluding locomotion-responsive neurons. (I) Averaged calcium traces of corresponding responsive neuron types in the opto-itch session. (J) Proportion of responsive neurons in the chloroquine and opto-itch session after excluding the locomotion-responsive neurons (*n* = 4 mice). Error bars, SEM. (K) Heat maps of the same neuronal population in the chloroquine and opto-itch session (*n* = 764 neurons). Neurons were rank-ordered by their response magnitude after the scratching onset in the chloroquine session. Each row represented responses from the same neuron to different itch stimuli. (L) The same example neuron reliably activated in both the chloroquine and opto-itch session.

Revealing the mechanism underlying perceptual detection relies on examining the neural dynamics that respond to the stimulus at the detection threshold [16,30]. However, it has been difficult to precisely control the pruritic input with chemical pruritic stimuli. We thus employed optogenetic stimulation to achieve precise control of pruritic stimuli, in place of chemical pruritogen application, which has a low temporal resolution [11,31] and poor control of stimulus intensity. This was achieved by optogenetic stimulation of spinal itch-selective gastrin-releasing peptide receptor-expressing (GRPR^+^) neurons [32]. We confirmed that chemogenetic activation of the spinal GRPR^+^ neurons induced S1Tr-dependent scratching behavior (Supplementary Fig. S3A and B), and chemogenetic inhibition of these neurons blocked itch signal processing (Supplementary Fig. S3C and D). To optogenetically activate spinal GRPR^+^ neurons, we injected AAV-Flex-ChrimsonR [33] into the dorsal horn of the cervical spinal cord of GRPR-iCreER mice, and implanted a red-light LED above the injection site for stimulation in free-moving mice (Fig. 3A–C; Supplementary Fig. S3E). In mice expressing ChrimsonR in spinal GRPR^+^ neurons, we found that optogenetic activation of these neurons evoked robust scratching behavior (Fig. 3D; Supplementary Fig. S3F; Supplementary Movie S2). The probability of scratching behavior increased with the intensity and frequency of the optogenetic stimulation (Fig. 3E; Supplementary Fig. S3G and H).

We next examined the dynamics of S1Tr neurons in response to optogenetic activation of spinal itch-selective neurons (abbreviated as ‘opto-itch’). We expressed GCaMP6s in S1Tr pyramidal neurons and ChrimsonR in spinal GRPR^+^ neurons via local injections of AAV (Fig. 3A, B and F; Supplementary Fig. S4A and B). We found that 17.4% (133/764) of recorded S1Tr neurons responded to opto-itch stimuli (5 Hz, 20 mW) with a high trial-to-trial reliability (Fig. 3G and J; Supplementary Fig. S4C–F), and 5.3% (7/133) of these responsive neurons (type II) peaked before the scratching onset, while a larger proportion (126/133 or 94.7%) of neurons (type I) showed sustained activity after the scratching onset (Supplementary Fig. S4G). Moreover, a large proportion (76/133 or 57.1%) of these responsive neurons exhibited elevated activity before the scratching onset (Fig. 3H and I; Supplementary Fig. S4H and I), in line with the above findings using pruritogens that a subpopulation of S1Tr pyramidal neurons encoded itch perception.

To determine whether the opto-itch stimulation activated the S1Tr neurons in a similar pattern to the chemical pruritogen, we examined the response of the same population of S1Tr neurons to both opto-itch and chloroquine (*n* = 764 neurons). A comparable proportion of S1Tr pyramidal neurons were activated near the onset of scratching behavior in response to these two different pruritic stimuli after excluding locomotion-responsive neurons (Fig. 3J and K). About one-quarter of the individual responsive neurons defined in the chloroquine or opto-itch session showed responses to both pruritic stimuli, and the response onset latency of these responsive neurons was comparable (Fig. 3L; Supplementary Fig. S4J and K). A large fraction of neurons responding to both pruritic stimuli (31/47 or 66.0%) exhibited congruent response latency relative to the scratching behavior onset under two different conditions (Supplementary Fig. S4L), supporting the notion that two types of pruritic stimuli evoked consistent activity profiles of S1Tr pyramidal neurons. Thus, opto-itch could mimic chemical pruritic stimulus, with better temporal resolution.

### Neuronal ignition of the primary somatosensory cortex during itch perception

**Figure 4.**
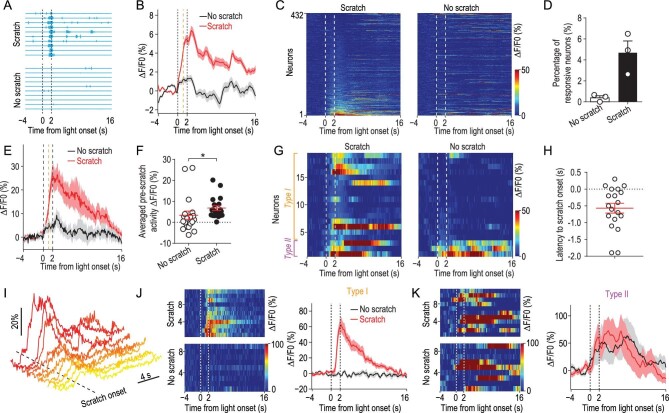
Activity of the primary somatosensory cortex in response to opto-itch stimulus at the threshold. (A) Behavioral traces divided into scratching or non-scratching trials of an example mouse (*n* = 3 mice) in response to the opto-itch stimulus at the threshold in an imaging session. Vertical dashed lines, light onset and offset. (B) Smoothed average calcium traces from all neurons (432 neurons from 3 mice) in two trial types. Orange vertical dashed line, averaged scratching onset from all trials. Shading represents SEM. (C) Population activity of all imaged neurons (*n* = 432) in scratching or non-scratching trials. Neurons were rank-ordered by their response magnitude after light onset in scratching trials. Each row represented responses from the same neuron in different trial types. (D) Percentage of responsive neurons to opto-itch stimulation in scratching or non-scratching trials (*n* = 3 mice) after excluding locomotion-responsive neurons. (E) Averaged calcium traces from responsive neurons defined in scratching trials by two trial types. Orange vertical dashed line, averaged scratching onset from all scratching trials. (F) Quantification of the averaged pre-scratch activity during optogenetic stimulation in different trial types from the responsive neurons (*n* = 19). Wilcoxon test. ^*^P < 0.05. Error bars, SEM. (G) Heat map showing the activity of the responsive neurons in scratching or non-scratching trials. Neurons exhibiting significant response to opto-itch stimulation in scratching trials but not in non-scratching trials were classified as type I, while those exhibiting responses in both trial types were classified as type II. (H) Distribution of the response latency to the scratching onset of responsive neurons in scratching trials (*n* = 19). (I) Calcium traces of responsive neurons to opto-itch stimulation in scratching trials from one FOV (*n* = 10). (J) Left: heat map of the calcium activity of one example type I neuron in scratching or non-scratching trials. Right: smoothed averaged calcium trace of this neuron. (K) Left: heat map of the calcium activity of one example type II neuron in scratching or non-scratching trials. Right: smoothed averaged calcium trace of this neuron.

To determine the activity pattern of S1Tr neurons during itch perception, we recorded the activity of S1Tr neurons in response to the opto-itch stimulation near the detection threshold, at which only about half of the trials successfully evoked the scratching behavior (Fig. 4A). We found that, in response to the same threshold opto-itch stimulus, S1Tr neurons exhibited stronger activity in scratching trials than non-scratching trials, and this difference already emerged prior to the onset of scratching (Fig. 4B). Notably, in trials in which the animal scratched, the threshold opto-itch stimulus reliably activated only ∼4.4% (19/432) of all recorded S1Tr neurons after excluding locomotion-responsive neurons (Fig. 4C and D), and these responsive neurons exhibited significantly stronger activity prior to scratching onset in scratching trials than non-scratching trials (Fig. 4E and F), suggesting that the generation of itch perception may only require the simultaneous activation of a very small population of S1Tr neurons. By contrast, very few neurons (0.5%, 2/432) were activated by the same opto-itch stimulus in trials without scratching, while the rest remained silent (Fig. 4C and D). These findings indicate that a subpopulation of S1 pyramidal neurons are recruited when itch perception is produced, while the failure to perceive itch sensation is reflected by the absence of significant S1Tr neuronal responses. This is in line with the neuronal ignition predicted by the global neuronal workspace hypothesis of consciousness, involving large-scale neuronal excitation resulting from recurrent excitation [34]. Importantly, this ignition-like activity could be discerned by differential activity patterns of individual neurons during scratching vs. non-scratching trials. Among neurons reliably responding to the threshold opto-itch stimulus in trials that scratching was evoked (*n* = 19 neurons), most (16/19, ‘type I’ neurons) of them showed no significant activity during the stimulus presentation period in non-scratching trials (Fig. 4G and J), thus these type I neurons exhibited an ‘all-or-none’ response pattern. In addition, most of the responsive neurons (16/19) increased their activity before the scratching onset, confirming the itch encoding property of these neurons (Fig. 4H and I). Only a few neurons (3/19, ‘type II’) showed comparable responses between these two different types of trials (Fig. 4G and K). This is reminiscent of visual-stimulus-evoked neuronal activity recorded from the human medial temporal lobe at the threshold of conscious visual recognition, where perception was reflected by an ‘all-or-none’ response pattern [35]. These data further suggest that S1 likely contributes to the generation of itch perception.

## DISCUSSION

In the mammalian brain, it is critical for external sensory information to reach the cortex in order to generate conscious perception. In this study, we examined the representation of itch perception in S1, and found that a subset of pyramidal neurons in layer 2/3 of S1 was capable of encoding itch perception. The itch-perception-associated neurons in S1 displayed an ignition-like activation pattern when an itch was perceived, similar to the response profile of cortical areas during visual perception [36].

Our study demonstrates that a fraction of S1 pyramidal neurons could encode the behavioral output of itch perception, as indicated by the scratching-predicting activity prior to behavioral onset (Fig. 2L–Q), suggesting that these neurons encode itch perception. This is in contrast to previous recording studies examining the itch-coding property of S1, the thalamus and the spinal projection neurons, as these electrophysiological experiments were conducted in anesthetized animals that could not behaviorally report the perception of itch [9–13]. The detection of perception-related activity in mouse S1 is consistent with previous studies showing that S1 neurons signal tactile perceptual choices and causally contribute to driving the subjective percept [17,18,37]. This is, however, different from previous studies in primates that have shown that perceptual judgement is generated by neurons downstream of S1 but not S1 [16,38]. The discrepancy in observations between studies might result from the dramatic differences in the brain organization of different animal species, as well as the diverse perceptual tasks [15]. Thus, it may not be possible to generalize the observation in mouse S1 to higher species such as primates. Nevertheless, it is possible that the perception-related activity in S1 reflects feedback and recurrent connections involving multiple brain areas [34,39,40], as the top-down projections from high-order areas including motor cortices have been shown to play essential roles in controlling and modulating perception [19,20,41,42], and S1 might be part of the neural network responsible for itch perception.

Our study revealed an ignition-like activation pattern of layer 2/3 neurons in S1 associated with itch perception. We found that, in response to the opto-itch stimulus at the threshold, a fraction of S1Tr neurons were activated during trials in which the animal scratched, while very few neurons were recruited in non-scratching trials (Fig. 4A–D), suggesting a selective activation of S1 pyramidal neurons during itch perception. At the population level, segregated S1 activity was detected via the observation that the same pruritic stimulus provoked different perceptual choices, which is similar to other somatosensory, as well as visual, perceptual studies [36,37]. Interestingly, we observed an ‘all-or-none’ response pattern in S1 neurons when an itch was perceived (Fig. 4G and J), similar to that observed in the mouse secondary somatosensory cortex in a whisker touch detection task, as well as in the medial temporal lobe of humans in response to a visual stimulus [35,37]. This is in contrast with previous studies showing that a smaller degree of activation in individual S1 neurons was still detected in trials when mice failed to report the presence of stimuli [17,18]. This difference could have resulted from varying degrees of animal training in different paradigms, since the learning process could cause plastic change of the property of S1 neurons [22,23]. In our study, no training was required for mice to report itch perception. This is a unique advantage of our paradigm, as the scratching response is natural, and the intrinsic property of S1 neurons to sensory stimuli would be retained. In the future, it will be important to determine whether a specific subclass of S1 neuronal population holds the information for itch perceptual detection, and which downstream brain regions receive the itch perceptual information conveyed by S1.

A relatively small proportion of S1 pyramidal neurons were recruited at the detection threshold of itch (Fig. 4C and D). The fraction of defined responsive neurons was slightly smaller than that in some previous studies examining the S1 responses to cooling or tactile stimulus [28,37], which could be caused by differential sensitivity of different body parts. In addition, we found that only about one-quarter of defined itch-responsive neurons overlapped across the opto-itch and chloroquine sessions, and the non-overlapping of individual responsive neurons in different itch models could be attributed to a few possible factors. One might be the big variability of the number of trials in the chloroquine session in different mice, which could result in certain contingency in defining the significant responsive neurons (Methods). Another reason might be the intrinsic sparseness of layer 2/3 S1 neuronal responses to sensory stimuli [43–45], which could lead to the recruitment of different neuronal populations in different sessions. Also, we cannot completely rule out the possibility that the artificial manipulation of spinal GRPR^+^ neurons also elicited other types of sensory input, which could also contribute to the non-overlapping S1 responses between opto-itch and chloroquine sessions.

We found that a subfraction of S1Tr neurons responded to locomotion, and some of these neurons showed increased activity before locomotion onset (Supplementary Fig. S2A–D), suggesting that these S1 neurons encoded motor-related information such as motor planning. Thus, these neurons were excluded when defining the itch-responsive neurons. However, we recognize that the movements involved in itch-induced scratching were presumably somewhat different from those involved in locomotion, and we cannot accurately filter the scratching-dependent motor responses from defined responsive S1 neurons. Besides, our results did not rule out the possibility that the activity of certain S1 pyramidal neurons reflected other information, including the anticipation of itch relief, as the activity of these neurons would also increase before the scratching behavior onset. Moreover, a substantial proportion of itch-responsive neurons showed progressively increased activity after scratching onset, suggesting that these itch-responsive neurons also responded to other somatosensory inputs associated with the scratching behavior [9,28]. Moreover, S1 could also adopt a population coding mechanism to selectively process itch information as suggested by a recent study [46]. Due to the close relationship between itch and pain, it is interesting to examine whether these two submodalities could activate distinct neuron populations in S1, and further experiments are warranted to directly test this. Nevertheless, the existence of non-locomotion-responsive itch-encoding neurons, as well as the fact that the activity of a subfraction of S1 pyramidal neurons could predict the scratching behavior strongly, supports the notion that a subfraction of S1 neurons have the capability to encode itch perception. Additional experiments, including the specific manipulation of itch-activated S1Tr neurons, are required to further test their role in itch perception.

This study established a new opto-itch paradigm for deciphering the neural mechanisms underlying itch processing with quantitative analysis. We showed that optogenetic stimulation of spinal itch-selective GRPR^+^ neurons evoked intensity-dependent itch-like scratching behavior, similar to the chemical itch model (Fig. 3D and E). The evoked scratching behavior showed high trial-by-trial reliability, which is highly suitable for investigating the mechanism underlying itch perception. This is in contrast to optogenetic activation of primary sensory neurons/fibers, which did not elicit scratching with high fidelity on a trial-by-trial basis [47,48]. Moreover, the response pattern of S1 neurons evoked by the opto-itch stimulation was comparable to that by pruritogens, thus the newly established opto-itch paradigm well mimicked the properties of chemical pruritus. The opto-itch paradigm allows for precise control of the timing and intensity of pruritic input, making it possible for quantitative analysis of itch perception, similar to the strategy used in the olfactory system [49]. This new paradigm complements chemical itch models, and offers an opportunity to directly measure the perceptual response during the natural itch–scratch cycle. The itch-coding S1 neurons identified in this study are mostly associated with acute chemical itch, as chloroquine is a pruritogen recognized in the histamine-independent pathway, while the opto-itch well mimics chloroquine itch. Although we speculate that the itch perception investigated here might be generalized to other itch models such as mechanical or chronic itch, further studies using other itch models are needed to directly test this hypothesis. On the other hand, the application of the newly developed mini-2P in free-moving mice [24] rendered it possible to directly measure the activity of the S1 neuronal population while the itch perception could be behaviorally reported with scratching, which is difficult to achieve with traditional *in vivo* two-photon imaging in head-fixed animals [20,50].

In summary, our study revealed the representation of itch in S1 at the cellular level, and demonstrated that layer 2/3 pyramidal neurons in S1 are capable of encoding itch perception. Importantly, our study also revealed the ignition-like activity pattern of S1 pyramidal neurons during itch perception. Thus, this study offered new insights and understanding with regard to itch perception in freely behaving animals.

## MATERIALS AND METHODS

Information on materials used to conduct the research, and methods used in the analysis, is available in the supplementary data.

## DATA AVAILABILITY

All relevant data and code for this study can be made available by the corresponding author upon reasonable request.

## Supplementary Material

nwab218_Supplemental_Files
