# Supplementary material for: Itch perception is reflected by neuronal ignition in the primary somatosensory cortex
Source: Natl Sci Rev. 2021 Dec 3;9(6):nwab218. doi: 10.1093/nsr/nwab218 (PMC9232292; doi:10.1093/nsr/nwab218)
Supplement: nwab218_Supplemental_Files [file nwab218_supplemental_files.zip › Supplementary_data_itch_perception_20211130.docx]

Supplementary data for

**Itch perception is reflected by neuronal ignition in the primary somatosensory cortex**

Xiao-Jun Chen^1,2^, Yan-He Liu^1,2^, Ning-Long Xu^1,2,3^, Yan-Gang Sun^1,3,^*

*Corresponding author. Email: yangang.sun@ion.ac.cn

**This PDF file includes:**

Materials and Methods

Figs. S1-S4

Movies S1-S2

Supplementary references

**Other Supplementary Materials for this manuscript include the following:**

Movies S1-S2

**Materials and Methods**

**Animals**

Male adult (7-14 weeks of age) C57BL/6N, GRPR-iCreER mice were used in all behavioral and imaging experiments. C57BL/6N wild type mice were purchased from SLAC laboratory (Shanghai). The generation of GRPR-iCreER mice has been described previously ([1](#_ENREF_1)). All mice were housed on a 12-hr light-dark cycle (lights on at 7:00 am) with ad libitum food and water. All procedures were approved by the Animal Care and Use Committee of the Center for Excellence in Brain Science & Intelligence Technology, Chinese Academy of Sciences, Shanghai, China (#NA-005-2019).

**Surgeries**

Mice were anesthetized with sodium pentobarbital solution (100 mg/kg body weight) for all surgeries, and the body temperature was maintained at 37 °C using a heating pad. Ophthalmic ointment was applied to maintain eye lubrication. All injections were performed using a picospritzer system controlled by a Master-8 stimulator. Mice were allowed to recover from anesthesia on a heating blanket before returning to their home cage. Mice were allowed at least two weeks to recover before behavioral and imaging experiments.

**Intra-spinal cord injection**

Paravertebral muscles and other tissues were retracted at vertebra level C1-C5, and the exposed vertebral column was mounted and fixed on a stereotaxic apparatus. One segment of vertebrae bone (C2 or C3) was removed, and a small incision was made on the dura for smooth penetration of the injection pipette. AAV-hSyn-Flex-ChrimsonR-tdTomato (AAV2/9, titer: 3.7x10^12^ v.g./ml), AAV-hSyn-DIO-hM4Di-mCherry (AAV2/8, titer: 5.4x10^12^ v.g./ml), AAV-hSyn-Flex-hM3D-mCherry (AAV2/9, titer: 4.3x10^12^ v.g./ml) or AAV-hSyn-DIO-mCherry (AAV2/9, titer: 3x10^12^ v.g./ml) was injected into the right side of the spinal cord at 3-4 injection sites (interspaced by 400-500 μm, ~400 nl per site) with a glass pipette targeted at the superficial dorsal horn. The pipette was withdrawn 5 min after the virus injection.

**LED implantation**

For mice used in spinal optogenetics experiments, a 630 nm LED (Teleopto) was placed above the virus injection area of the exposed spinal cord, and was stabilized with 3M Vetbond and dental cement. The surrounding skin was closed with stitches for better stability.

**Chronic imaging window**

A circular craniotomy (~3 mm in diameter) was performed over the left S1Tr (center coordinate: AP +1.45 mm; ML +1.85 mm), and the dura was left intact. AAV-CaMKII-GCaMP6s (AAV2/9, titer: 1.23x10^13^ v.g./ml) was slowly injected (250 nl per site, 2 sites interspaced by 250 μm; ~350 μm beneath cortical surface) into the cortex. The injection pipette was withdrawn 5 min after the end of the injection. An imaging window constructed with two glass coverslips (inner layer diameter ~3 mm, outer layer diameter ~5 mm) joined with an ultraviolet curable optical glue was inserted into the craniotomy, sealed in place with dental cement.

**Muscimol inactivation**

About 400 nl of either GABAA agonist muscimol (Sigma, 1 mg/ml) or sterilized PBS was injected in the left S1Tr (center coordinate: AP +1.45 mm; ML +1.85 mm; DV -0.4 mm) or left V1 (center coordinate: AP +4.0 mm; ML +2.70 mm; DV -0.4 mm) after anesthesia, and we waited for 5 minutes at the end of the injection before withdrawing the pipette. Behavioral experiments were conducted 90-120 min after the injection. We injected 400 nl fluorescent muscimol bodipy (Invitrogen, 2 mg/ml) in S1Tr or V1 to identify the diffusion range of muscimol before perfusion.

**Pharmacogenetic inactivation**

At least 3 weeks after spinal injection of AAV-hSyn-DIO-hM4Di-mCherry or AAV-hSyn-DIO-mCherry, GRPR-iCreER mice were used for behavioral tests. These mice received Clozapine N-oxide (CNO, 1 mg/kg, i.p.) injection 30 min prior to pruritogen application, and the scratching behavior was then recorded for 40-45 min.

**Pharmacogenetic activation**

At least 3 weeks after spinal injection of AAV-hSyn-Flex-hM3Dq-mCherry or AAV-hSyn-DIO-mCherry, GRPR-iCreER mice were used for behavioral tests. These mice received Clozapine N-oxide (CNO, 1 mg/kg, i.p.) application 90-120 min after muscimol injection, and the scratching behavior was then recorded for 60 min.

**Tamoxifen injection**

Tamoxifen was dissolved in sunflower oil at the concentration of 20 mg/ml, and was injected in GRPR-iCreER mice at the dose of 150 mg/kg (i.p.) body weight for five consecutive days after virus injection.

**Itch behavioral test**

For automatic measurement of scratching behavior, a small (1 mm in diameter, 3 mm long cylinder) magnet was implanted into the back of the right hindpaw for each mouse, and the scratching behavior was recorded with a magnetic induction method as described previously ([1](#_ENREF_1)). For optogenetic stimulation, the stimulus was delivered through a wireless optogenetic system (Teleopto). Adjustment of light frequency and LED output power was achieved by a Master-9 stimulator. At least 8-10 trials of each intensity were acquired. The scratching behavior and detailed measurement was analyzed using custom-written codes in Matlab with manual adjustment as described previously ([1](#_ENREF_1)). Scratching behavior consisted of a burst of current fluctuations exhibiting periodic characteristics, with each cycle corresponding to one hindpaw movement, referred hereafter as “a scratching spike”. Several continuous scratching spikes constituted a scratching bout ([1](#_ENREF_1)). A cluster of scratching bouts with the interval less than 2 seconds was defined as a scratching train, and only scratching trains without other scratching events during a minimal 10-s time window both before and after this train were considered as clean scratching trains in the session with chloroquine application.

In response to the optogenetic stimulation of spinal GRPR^+^ neurons, the evoked scratching behavior was defined as the scratching trains whose onset was within the 2-s LED illumination period, but not the spontaneous scratching trains with their onset prior to stimulation onset or later than stimulation offset. At the stimulation intensity of 5 Hz, 20 mW (2 s), scratching behavior was not always evoked in some mice. For 3 mice, about half of the stimulation trials successfully evoked scratching behavior at this intensity. These animals were used for analysis of the activity of S1Tr neurons in response to the opto-itch stimulation near the detection “threshold”.

**Immunohistochemistry**

After behavioral or imaging experiments, mice were used for immunohistochemistry. Mice were anesthetized, perfused transcardially with 4% paraformaldehyde (PFA, Sigma) solution. LEDs and head plates were retrieved for recycling use. Spinal cord and brains were dissected, post-fixed overnight at 4 °C in 4% PFA, then cryoprotected in 30% sucrose in PBS at 4 °C. 40 μm free floating sections were prepared using Leica CM 1950. The sections were first washed in PBS, then blocked with 5% normal donkey serum in PBST (0.3% Triton X-100) at room temperature for 30-60 min. After incubation of primary antibodies at 4 °C overnight, and secondary antibodies at room temperature for 2 hours, the sections were mounted on slides for imaging. Primary antibody used in IHC was goat anti-Iba1 (1:500, Abcam, ab5076). Secondary antibodies were donkey anti-goat IgG-Alexa 488 (1:200, Jackson, 705-546-147). Images were taken using Olympus VS120 fluorescence microscope.

**In vivo two-photon calcium imaging in free-moving mice**

The imaging experiment is performed at Nanjing Brain Observatory (PKU-Nanjing Joint Institute of Translational Medicine), and the procedure was conducted as described previously ([2](#_ENREF_2)). Briefly, after habituation, the mice were restrained with a holder tube to confirm the virus expression and imaging field. With the switchable platform, we identified the fluorescent ROIs with the benchtop microscope first and then mounted the fast high-resolution, miniaturized two-photon microscope (mini-2P) to the designated region. Next, we glued a headpiece to the plate which had been fixed to the skull during craniotomy, and 1.5% low-melting point agarose was applied to cover the chronic window before imaging. All mice were habituated for one week after headpiece implantation, and the headpiece was covered with parafilm and plasticine between sessions to keep the chronic window clean. The mouse hair was shaved to expose the rostral back skin for delivery of pruritogens. Images (512 × 512 pixels, 400 × 400 μm) were acquired at ~10 Hz using National Instruments software continuously.

For imaging sessions with chloroquine application, images were collected for 45-60 min after intradermal injection of chloroquine (200 μg/ 50 μL) in the rostral back skin. For optogenetic stimulation, the spinal implanted LED was connected to a Master-9 stimulator via custom-made wires. For each trial, a 2-s LED illumination (5 Hz 20 mW) was delivered after a 2-s baseline period, ISI of the optogenetic stimulation of spinal GRPR^+^ neurons was set to 20 s, about 20 consecutive trials were sampled.

**Imaging data analysis**

Lateral drifts in two-photon images was corrected by custom-written algorithm. All remaining processing and analysis was performed by custom-written Matlab codes. ROIs corresponding to visually identifiable soma were then automatically outlined. The fluorescence of each cell body was measured by averaging the pixels within the ROI, with a correction for neuropil contamination, as previously stated ([3](#_ENREF_3)). Briefly, the fluorescence of a soma was estimated as F_cell_true_(t) = F_cell_measured_(t) – 0.7×F_neuropil_(t). The neuropil signal F_neuropil_(t) surrounding each soma was measured by averaging the signal of all pixels within a 10-pixel radius from the cell center (excluding selected cells).

For mini-2P imaging sessions with chloroquine application, only clean scratching trains defined in the itch behavioral test section were used for further analysis, since the S1 calcium activity could be influenced by nearby scratching behavior. F0 was determined by the average of the 4-s period (-6 to -2 s) prior to the scratching onset, and the s.d. of the baseline period was calculated. Neurons were categorized as scratch-responsive when the mean ΔF/F of the response window (0 - 4 s from the scratching onset) was over 1 × s.d. higher than baseline in at least 50% individual trials (trains), and the averaged ΔF/F of the response window from all trials was at least 2 × s.d. higher than averaged baseline activity from all trials. The response magnitude was determined as the mean activity of a 6-s period after the scratching onset. For each neuron, trials with mean activity of the response window being over 1 × s.d. higher than baseline were considered as the responsive trials, trials without this level of activation were considered as non-responsive trials, and the trial-by-trial reliability was calculated by the number of responsive trials divided by the total trial number. For each responsive neuron, the response onset was defined as the beginning of the first 5 successive sample points higher than 2 × s.d. of baseline activity in the averaged trace from all trials (trains), and the response latency was determined by subtracting the pre-scratch duration (6 s) from the onset time. The behaviorally quiet period was generated by removing the epochs spanning from 10 seconds prior to scratching to 10 seconds after scratching from the original behavioral trace. The temporal correlation between neurons was determined by calculating pair-wise Pearson’s Correlation Coefficient between the activity of neuron pairs after normalizing each neuron’s activity to peak during the sample period, and control neurons were picked by randomly sampling the same number of neurons as responsive neurons from the same FOV.

For locomotion analysis in chloroquine sessions, only clean locomotion events without scratching during a minimal 20-s time window both before and after this locomotion trial were used for further analysis. F0 was determined by the average of the 4-s period (-6 to -2 s) prior to the locomotion onset, and the s.d. of the baseline period was calculated. Neurons were categorized as locomotion-responsive when the mean ΔF/F of the response window (0 - 4 s from the locomotion onset) was over 1 × s.d. higher than baseline in at least 50% individual trials (locomotion events), and the averaged ΔF/F of the response window from all trials was at least 2 × s.d. higher than averaged baseline activity from all trials. Neurons were considered as itch-specific responders when a given neuron was defined as responsive to chloroquine, while not categorized as locomotion-responsive in the same session.

For mini-2P imaging sessions with the opto-itch stimulation, the neuronal activity was aligned to scratch or stimulus onset. When aligned to scratch, F0 was determined by the average of the 2-s period (-4 to -2 s) prior to the scratching onset, and the s.d. of the baseline period was calculated. Neurons were categorized as scratch responsive when the mean ΔF/F after the scratching onset (0 - 4 s from the scratching onset) was over 1 × s.d. higher than baseline in at least 50% individual trials, and the mean ΔF/F after the scratching onset was at least 2 × s.d. higher than baseline averaged from all trials. Neurons were considered as opto-itch-specific responders when a given neuron was defined as responsive to opto-itch, while not categorized as locomotion-responsive in the chloroquine session from the same mice. The response magnitude was determined as the mean activity of a 6-s period after the scratching onset. For each responsive neuron, the response onset was defined as the beginning of the first 5 successive sample points higher than 2 × s.d. of baseline activity in the averaged trace from all trials, and the response latency was determined by subtracting the pre-scratch duration (4 s) from the onset time. For each reliable responsive neuron in chloroquine and opto-itch session, the response latencies in both sessions were summarized, and the stability of response latency was determined by dividing the number of neurons within each latency category (e.g., ≤ 0 in chloroquine session & > 0 in opto-itch session) by the total number of reliable responsive neurons in both sessions.

In the case of stimulus alignment in the opto-itch session, F0 was determined by the average of the 4-s period (-4 to 0 s) prior to stimulus onset, and the s.d. of the baseline period was calculated. Neurons were categorized as stimulus responsive when the mean ΔF/F after stimulation onset (0 - 4 s from stimulus onset) was over 1 × s.d. higher than baseline in at least 50% individual trials, and the mean ΔF/F after stimulus onset was at least 2 × s.d. higher than baseline averaged from all trials. To quantify the pre-scratch activity during opto-itch stimulation for each neuron, we calculated the mean activity during the epoch from stimulation onset to the averaged onset of scratching behavior. To obtain the smoothed averaged trace, we smoothed the calcium data by a moving average filter with a span of 5 sample points (~0.5 s). The neurons with the “all-or-none” response pattern was defined as the neurons showing significant responses to the threshold opto-itch stimulation in scratching trials, while not showing significant responses in non-scratching trials.

For single neuron ideal observer analysis in the chloroquine session, we used receiver operating characteristic (ROC) analysis. Mean calcium activity of the S1Tr pyramidal neurons within the 2 s prior to clean scratching trains were compared with the mean activity within a 2-s period in the middle of a behaviorally quiet period (> 40 s without scratching). ROC curve was generated by plotting the fraction of scratch trials in which the response exceeded criterion against the fraction of scratch-absent trials in which the response exceeded criterion for all criterion levels across both trial types. The area under the ROC curve (AUC) characterizes the discriminability of scratch trials from blank trials. To determine the statistical significance, a null ROC value distribution was computed by randomly assigning the neuronal responses to the relevant trial type. This process was repeated 1000 times to obtain a null distribution of AUC values. For each neuron, the 95th percentile of the shuffled AUC values was defined as the threshold for significant discrimination. For each neuron, the normalized activity was determined by dividing the ΔF/F value by the peak ΔF/F in the averaged trace from all trials. The peak time was determined as the first of 10 successive sample points (~1 s) with highest activity in the averaged trace. Neurons that peaked after the scratching onset were categorized as type I, while neurons peaked before the scratching onset were categorized as type II.

**Statistical Analysis**

Statistical analyses were performed using GraphPad Prism or Matlab, and all test methods and results are listed in figure legends. All results are expressed as means ± SEM, and P < 0.05 is considered as significant. All tests were two tailed unless otherwise specified.

Supplementary figures

Fig. S1. Validation of the free-moving two-photon calcium imaging. (A) Schematic illustrating the microinjection of muscimol (400 ng/400 nL) in the contralateral S1Tr. (B) Diffusion of muscimol in the contralateral S1Tr. Scale bar, 500 μm. (C) Schematic depicting the muscimol diffusion area in S1Tr along the longitudinal axis from one mouse. (D) Inactivation of the contralateral S1Tr significantly impaired chloroquine-induced scratching behavior (n = 7 mice for each group). Mann-Whitney test. ** P < 0.01. Chloroquine, 200 μg/ 50 μL. (E-F) Inactivation of the contralateral S1Tr did not affect the traveling distance (E) or running velocity (F) in the open field test (n = 7 mice for each group). Mann-Whitney test. (G) Schematic illustrating the microinjection of muscimol (400 ng/400 nL) in contralateral V1. (H) Diffusion of muscimol in contralateral V1. Scale bar, 500 μm. (I) Schematic depicting the muscimol diffusion area in V1 along the longitudinal axis from one example mouse. (J) Inactivation of contralateral V1 did not significantly affect chloroquine-induced scratching behavior (n = 7 mice for each group). Mann-Whitney test. (K) Schematic of AAV-CaMKII-GCaMP6s injection in the contralateral S1Tr. (L) Graph showing image of one example FOV for the 5th, 15th, and 30th min of consecutive 10-Hz imaging after intradermal injection of chloroquine. Scale bar, 100 μm. (M) Distribution of lateral shift on a frame-by-frame basis in an imaging session after intradermal injection of chloroquine. (N) Relative lateral shift of the FOV in (L) at corresponding time points. (O) Relative changes of the fluorescence in individual neurons (gray) and all neuron average (red) from the FOV in (L) at corresponding time points. (P) Lateral shift around the offset of scratching behavior relative to the scratching onset in response to chloroquine during imaging. Shading represents SEM. (Q) The scratching behavior induced by intradermal injection of chloroquine in mice carrying the mini-2P as well as control mice without the head-mount. (R-U) Quantification of the scratching bout number (R), scratching train number (S), scratching bout duration (T) and spikes per scratching bout (U) in response to intradermal injection of chloroquine in two groups of animals (n = 5-8 mice for each group). Mann-Whitney test. n.s., nonsignificant. Error bars, SEM.

Fig. S2. S1Tr responded to a chemical pruritic stimulus. (A) Behavioral traces of locomotion trials in a chloroquine session. (B) Heat map showing the calcium activity of one neuron during corresponding locomotion trials in (A). (C) Averaged calcium trace of the neuron in (B) corresponding to all locomotion trials. (D) Heat map showing the activity of all imaged neurons in locomotion trials. Neurons were rank-ordered by their response magnitude after the locomotion onset. (E) Proportion of responsive neurons in the chloroquine session after excluding locomotion-activated neurons. (F) Cumulative distribution of trial-by-trial reliability of S1Tr responses to chloroquine-induced scratching behavior for all imaged neurons. (G) Distribution of responsive neurons in one FOV from the chloroquine session. Colored dots, neurons showing positive responses. Scale bar, 100 μm. (H) Cumulative distribution of pair-wise distances among neurons activated in the chloroquine session, as well as all imaged neurons in S1Tr (2560 responsive and 86686 all-neuron pairs). Kolmogorov-Smirnov test. (I) Schematic depicting the sampling of a behaviorally quiet period without scratching from the original behavioral trace. (J-K) Heat map of responsive neurons with differential response latency in the chloroquine session. Neurons were rank-ordered by their response magnitude after the scratching onset. (L) Behavioral traces of an example mouse in response to intradermal injection of chloroquine aligned to the scratching offset of individual trains in an imaging session. (M) Heat map of the calcium activity of one example neuron to corresponding scratching trains in (L). (N) Averaged calcium traces of the neuron in (M) corresponding to all trains aligned to the scratching offset. Shading represents SEM. (O) Heat map of all imaged neurons from 5 FOVs (n = 906 neurons) aligned to the scratching offset during chloroquine induced scratching behavior. Neurons were rank-ordered by their response magnitude after the scratching onset. (P) Distribution of the peak latency relative to the scratching offset of all responsive neurons with the response latency ≤ 0 s (n = 64 neurons from J). (Q) Response pattern of one representative neuron in the ROC analysis. ROC_insig, neuron not significantly discriminating two different trial types (scratch vs. blank control).

Fig. S3. Establishment of opto-itch model by optogenetic activation of spinal GRPR^+^ neurons. (A) Schematic illustrating the intraspinal injection of AAV-Flex-hM3Dq-mCherry in the right cervical spinal cord of GRPR-iCreER mice, followed by the microinjection of muscimol (400 ng/400 nL) in the contralateral S1Tr. (B) Inactivation of the contralateral S1Tr significantly impaired the scratching behavior induced by chemogenetic activation of spinal GRPR^+^ neurons (n = 8 mice for each group). CNO, 1 mg/kg. Mann-Whitney test. (C) Schematic of intraspinal injection of AAV-DIO-hM4Di-mCherry or AAV-DIO-mCherry in the cervical spinal cord of GRPR-iCreER mice. (D) Chemogenetic inactivation of cervical spinal GRPR^+^ neurons by CNO application (1 mg/kg, i.p.) decreased scratching behavior induced by chloroquine and histamine (n = 7-9 mice). Two-way ANOVA with Sidak’s multiple comparisons. Chloroquine, 200 μg/50 μL. Histamine, 500 μg/ 50 μL. ** P < 0.01. *** P < 0.001. (E) Expression of Iba1 in the cervical spinal sections of GRPR-iCreER mice receiving AAV-Flex-ChrimsonR-tdTomato injection in the right spinal cord. Scale bar, 200 μm. (F) The output power of the 630 nm LED under different triggering voltages at a 5 Hz pulse mode. Red bar, LED on period. (G) Example behavioral traces for scratching induced by optogenetic stimulation of spinal GRPR^+^ neurons at different intensities. Red bar, opto-itch stimulation. (H) Quantification of the latency to the scratching onset, and the scratching duration in response to optogenetic stimulation of spinal GRPR^+^ neurons at different intensities (n = 8 mice).

**Fig. S4. S1Tr neurons faithfully responded to opto-itch stimulus.** (**A**) Graph showing image of one FOV from the same mouse in the mini-2P sessions in response to chloroquine or opto-itch. Scale bar, 100 μm. (**B**) Relative changes of the fluorescence in individual neurons (gray) and all neuron average (red) from the FOV in (A) in corresponding imaging sessions. (**C**) Heat map of all imaged neurons from 6 FOVs (n = 1038 neurons) during the scratching behavior in response to opto-itch stimulation. Neurons were rank-ordered by their response magnitude after the scratching onset. (**D**) Behavioral traces of one mouse during opto-itch stimulation. (**E**) Heat map showing the calcium activity of one neuron aligned to corresponding scratching trains in (D). (**F**) Averaged calcium trace of the neuron in (E) in response to opto-itch stimulation. Shading represents SEM. (**G**) Normalized population activity of all responsive neurons defined in the opto-itch session (n = 133 neurons from 4 mice). Neurons were ordered by the peak time. Neurons showing sustained activity with peak occurring after the scratching onset were classified as type I, while those showing transient activity with peak occurring prior to the scratching onset were classified as type II. (**H**) Composition of all imaged neurons (n = 764 neurons from 4 mice) in the opto-itch session. (**I**) Averaged calcium trace of different types of responsive neurons aligned to the scratching offset. (**J**) Diagram showing the overlap in distribution of responsive S1Tr neurons (out of 764 neurons from 4 mice) to different itch stimuli. (**K**) The latency to response onset of the same individual responsive neurons (n = 47 neurons) to two different itch stimuli. Error bars, SEM. (**L**) Proportion of reliable responsive neurons that exhibited diverse response latency in the chloroquine and opto-itch sessions.

Movie S1. In vivo two-photon calcium imaging in S1Tr during chloroquine induced scratching behavior. Left, the behavioral recording and the real-time scratching trace of an example mouse during a 100-s period after intradermal injection of chloroquine. Right, an example FOV under the mini-2P during the corresponding period from the same mouse on the left, as well as the calcium transients of one example neuron during this period. The video was displayed at 2x speed.

**Movie S2. Optogenetic stimulation of spinal GRPR^+^ neurons evoked robust scratching behavior.** The behavior recording of an example mouse expressing ChrimsonR in spinal GRPR^+^ neurons in response to a red LED stimulation (20 Hz, 20 mW, 2 s) through a wireless optogenetic system. The scratching behavior faithfully followed the light stimulus at this intensity.

Supplementary references

1. Mu D, Deng J, Liu KF, et al.; A central neural circuit for itch sensation. *Science* 2017;**357**(6352):695-699. doi: 10.1126/science.aaf4918.

2. Zong W, Wu R, Li M, et al.; Fast high-resolution miniature two-photon microscopy for brain imaging in freely behaving mice. *Nat Methods* 2017;**14**(7):713-719. doi: 10.1038/nmeth.4305.

3. Chen TW, Wardill TJ, Sun Y, et al.; Ultrasensitive fluorescent proteins for imaging neuronal activity. *Nature* 2013;**499**(7458):295-300. doi: 10.1038/nature12354.
